# The Association of Myocardial Infarction History and Geriatric Syndromes in the Elderly: Data from the Cross-Sectional Study EVKALIPT

**DOI:** 10.3390/jcm13216420

**Published:** 2024-10-26

**Authors:** Vadim Zakiev, Natalya Vorobyeva, Irina Malaya, Yulia Kotovskaya, Olga Tkacheva

**Affiliations:** Russian Gerontology Clinical Research Center, Ministry of Healthcare of the Russian Federation, Pirogov Russian National Research Medical University, 129226 Moscow, Russiamalaya_ip@rgnkc.ru (I.M.); kotovskaya@bk.ru (Y.K.); tkacheva@rgnkc.ru (O.T.)

**Keywords:** cardiovascular diseases, elderly, frailty, geriatric syndrome, myocardial infarction history

## Abstract

**Background/Objectives**: In recent decades, the number of patients with chronic cardiovascular diseases (CVDs) has increased, and CVD survivors are more likely to be old and frail and to have multiple comorbidities. A better understanding of geriatric conditions and their prevalence would help improve the management of older patients with CVDs. The main objective of this study is to estimate the association of myocardial infarction (MI) history with geriatric syndromes (GSs) in people 65 years of age and older. **Methods**: The cross-sectional study EVKALIPT included patients who were 65 years of age and older. All patients underwent a comprehensive geriatric assessment. The presence of MI history was assessed by medical records. **Results**: A total of 4295 participants were included. The prevalence of MI history was 12.6%. According to univariate regression analysis, MI history was associated with an increase in the odds of 12 GSs by 1.3–2.4 times. Multivariate regression analysis showed that male sex and four GSs (impairment in basic and instrumental activities of daily living, depression, falls) were independently associated with a history of MI, with the odds ratio ranging from 1.28 to 1.86. **Conclusions:** This study showed the association between MI history and GSs.

## 1. Introduction

The global population is aging, and the proportion of elderly people is rising worldwide. It is expected that this proportion will be at least 22% by the middle of the twenty-first century [1]. Ischemic heart disease is the most important cause of death in elderly people, with thousands of years of life lost [2]. Frailty is a common geriatric syndrome (GS) and can be defined as a progressive age-related deterioration in physiological systems that results in extreme vulnerability to stressors and increases the risk of a range of adverse outcomes, including care dependence and death [3,4]. Prefrailty is defined as a clinically silent process that predisposes individuals to frailty [5]. The prevalence of frailty in high-income countries is around 4% at age 50–64 years, increasing to 17% in people of 65 years and older [6]. According to a recent meta-analysis, the estimated global prevalence of frailty is 12% and of prefrailty is 46% [7].

In the 1990s, most patients with cardiovascular diseases (CVDs) died of cardiac causes. Since then, modern treatments and interventions have increased the survival of patients with acute myocardial infarction (MI), and a decrease in cardiovascular mortality with an increase in non-cardiovascular mortality has been observed in subjects with MI [8]. As a result, the number of patients with chronic CVDs has increased, and CVD survivors are more likely to be older and frail with multiple comorbidities [9]. It is suggested that a better understanding of geriatric syndromes and their prevalence in MI survivors would help to improve the management of older patients with CVDs [10].

An epidemiological study of the prevalence of geriatric syndromes and age-associated diseases in elderly patients in regions of the Russian Federation with different climatic, economic, and demographic characteristics (EVKALIPT) was designed as a cross-sectional study and was performed as the initiative of the Russian Association of Gerontologists and Geriatricians and the Russian Gerontological Clinical Research Center (RGCRC) in cooperation with the National Research University Higher School of Economics. This study enrolled patients aged 65 years of age and older who lived in 11 regions of Russia. The primary aim of this study was to assess the prevalence of age-associated diseases, frailty, GSs, and comorbidities, as well as to conduct an analysis of their contribution to general health and functional status. The current article represents the subanalysis of the EVKALIPT study, which assess GSs and frailty among MI survivors. The study protocol and basic characteristics of the participants were described previously [11].

## 2. Materials and Methods

This study was performed from April 2018 to October 2019. The study inclusion criteria were an age of 65 years or older and signed informed consent to participate in the study.

All patients underwent a comprehensive geriatric assessment (CGA) based on a specially designed questionnaire and physical examination. CGA was carried out simultaneously by a geriatrician and a geriatric nurse at the patient’s location or residence. The questionnaire consisted of several modules about socio-economic status, occupational history, risk factors for chronic diseases, chronic diseases, drug therapy, obstetrics and gynecological history, falls and risk of falls, chronic pain, sensory deficits, oral health, urine and fecal incontinence, and use of aids. The questionnaire also included information about laboratory examination results and standard scales: the 15-item Geriatric Depression Scale (GDS-15), Basic Functional Activity Scale (Barthel Index), Lawton Instrumental Activities of Daily Living (IADL) Scale, Mini Nutritional Assessment (MNA) short-form, and Visual Analog Scale (VAS). These scales were used for the self-assessment of quality of life, health status, and intensity of pain syndrome at the examination and within the previous 7 days.

Physical examination included short physical performance battery (SPPB) tests, dynamometry, measurement of gait velocity, Mini-Cog test, measurement of height and body weight, calculation of body mass index (BMI), measurement of blood pressure (BP) and heart rate, and orthostatic test. The presence of chronic diseases (in particular, a previous myocardial infarction) was assessed by the patients’ medical records: outpatient charts, discharge summaries, and laboratory and instrumental examinations’ protocols.

This study was carried out in accordance with the principles of the Helsinki Declaration on Human Rights. This study was approved by the Local Ethics Committee of the RGCRC (protocol № 19). All patients signed an informed consent to participate in the study and to process personal data.

Statistical data analysis was performed using IBM^®^ SPSS^®^ Statistics version 23.0 (SPSS Inc., Chicago, IL, USA). The type of distribution of the quantitative variables was analyzed using the one-sample Kolmogorov–Smirnov test. In the case of a normal distribution, the data were presented as M ± SD, where M is the mean and SD is the standard deviation; in the case of a non-normal distribution, the results were presented as Me (25%; 75%), where Me is the median and 25% and 75% are the 25th and 75th percentiles. Qualitative ordinal variables are presented as Me (25%; 75%). Missing values were not filled in. For intergroup comparisons, Student’s *t*-test, the Mann–Whitney, Pearson’s χ^2^ tests, as well as Fisher’s two-tailed exact test were used. Relationships between variables were assessed using binary logistic regression by calculating of the odds ratio (OR) and 95% confidence interval (CI). Multivariate regression analyses were performed after adjusting for age and gender. We analyzed the variables using the direct stepwise selection method (step-by-step selection criteria: inclusion in the model at *p* = 0.05; removal from the model at *p* = 0.1); missing values were removed row by row. Differences were considered statistically significant with a two-sided *p* value < 0.05.

## 3. Results

This study included 4308 patients (30% men) aged 65–107 years. The majority of participants (60%) were examined in an outpatient setting, 20% were examined in a hospital, 19% were examined at home, and 1% were examined in residential institutions/assisted-living facilities. The main demographic, anthropometric, and subjects’ clinical characteristics according to age groups were presented previously [12].

**Figure 1:**
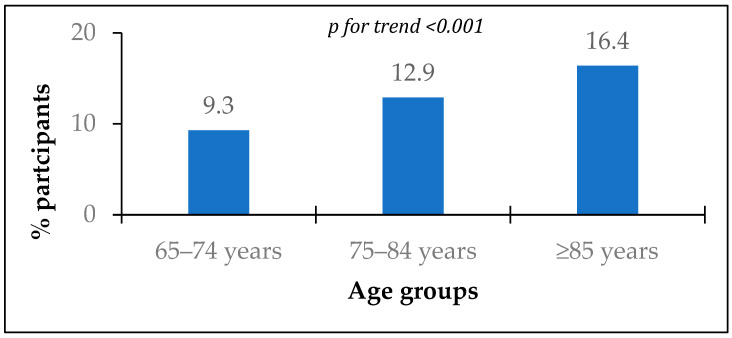
Prevalence of myocardial infarction history according to age groups.

Information about chronic diseases was available for 4295 participants. The prevalence of MI history was 12.6% and significantly increased with age (Figure 1).

Patients with a history of MI were on average 2 years older with a predominance of men (Table 1). Although there were no significant differences in BMI, patients with MI history had a body mass deficit 2.4 times more than people without MI history. There were more people with increased systolic and diastolic BP among patients with a history of MI.

Patients with a history of MI had worse geriatric status (Table 2). They had lower walking speed and hand grip strength. The proportion of subjects with decreased walking speed and hand grip strength was higher in patients with prior MI. They also had fewer points on the Barthel Index, Mini-Cog test, Lawton IADL Scale, MNA short-form, and SPPB. Patients with MI history had more points of GDS-15 and assessed their quality of life and health status lower and pain intensity higher than patients without a history of MI.

Patients with a history of MI used assistive devices more often, and the number per patient of such devices was significantly higher than in patients without MI (Table 3). Patients with prior MI were significantly more likely to use glasses, hearing aids, dentures, absorbent clothing, and mobility aids but not orthopedic devices.

The frequency of various GSs in patients with a history of MI was higher compared with patients without prior MI (Table 4). The most common GSs in patients with a history of MI were chronic pain syndrome (91%), basic (72%) and instrumental (72%) activities of daily living impairment in daily activities, frailty syndrome (70%), cognitive impairment (69%), depression (58%), and urinary incontinence (51%). The least common GSs were visual impairment (6.5%), fecal incontinence (5.6%), and bedsores (3.9%).

Univariate regression analysis was used to assess the relationship between a history of MI and GSs. Twelve GSs were sequentially considered dependent variables, while MI history was an independent variable. Univariate regression analysis demonstrated that MI history was associated with an increase in the OR of GSs by 1.3–2.4 times (Table 5).

The multivariate regression analysis included 12 GSs with a significance level of *p* < 0.05 according to the univariate regression analysis. MI history was considered a dependent variable; age (as an extended variable), sex, and 12 GSs were considered independent variables. Multivariate analysis showed that male sex and four GSs were independently associated with a history of MI, with the OR ranging from 1.28 to 1.86 (Table 6). The order of variables’ inclusion in the model was as follows: impairment in IADL, male sex, falls during the previous year, depression, and impairment in basic activities of daily living.

## 4. Limitations

First of all, this study was not originally designed to investigate the association between MI history and GSs, so we did not count the year of MI. That is why it is impossible to estimate what comes first: whether the development of GSs contributes to the development of MI or vice versa. Secondly, information about revascularization and myocardial systolic function was not collected in our study.

## 5. Discussion

The current subanalysis of the EVKALIPT study showed that the prevalence of MI history among elderly people (≥65 years) in Russia was 12.6% and significantly increased with age, while based on the study of Shalnova et al., the prevalence of MI among younger people (<65years) in Russia is 2.9% [13]. According to a recent meta-analysis, the global prevalence of MI in individuals <60 years is 3.8% and 9.5% in individuals >60 years old [14]. Thus, our study demonstrated a higher prevalence of MI among Russian elderly people than it is estimated in the world and younger populations.

The EVKALIPT study found associations between prior MI and different GSs. Univariate regression analysis showed MI history associations with frailty, depression, cognitive impairment, urinary incontinence, impairment in basic and IADL, sensory deficit and hearing loss, falls, chronic pain, malnutrition, and bedsores. However, the multivariate regression analysis adjusted by age and sex showed associations with only four GSs: impairment in basic and IADL, depression, and falls during the previous year. Patients with prior MI were also more likely to require assistive devices.

The literature data support the results of our study. People with cardiovascular diseases may have a high prevalence of frailty. For example, the prevalence of frailty among acute coronary syndrome (ACS) patients is 32% (95% confidence interval: 25–39%) [15]. Conversely, the prevalence of cardiovascular diseases is much higher in frail than non-frail people [16,17]. Frailty is associated with a significantly greater mortality risk irrespective of the type of cardiovascular disease and duration of follow-up [18]. There are many data about the negative impact of frailty and prefrailty on all-cause mortality and readmission in patients with ACS [15,19,20,21]. Baseline IADL impairment was also independently associated with incident acute myocardial infarction and all-cause mortality [22]. According to Nguyen TV et al., among patients undergoing chronic hemodialysis, the prevalence of impairment in basic and IADL, frailty, and malnutrition was higher in patients with CVDs [23].

The exact mechanisms of these associations are still unclear. Frailty is one of the most common GSs, while comorbidity is one of the key components of frailty. For example, a widely used deficit accumulation frailty model developed by K. Rockwood et al. includes different comorbidities like hypertension, chronic heart failure, ischemic heart disease, stroke, etc. Some versions of the Rockwood model include MI history as a separate factor [24], while others do not [25]. At the same time, MI is one of the most common reasons for heart failure, which is part of the deficit accumulation model in all its versions. Our data indicate that it is necessary to consider history of MI when assessing the Rockwood fragility index in all cases as there was an association of MI history with GSs in our study.

Another possible reason for association between MI history and GSs is overlapping pathogenetic mechanisms, one of which is inflammation. Most older individuals develop inflammageing, a condition characterized by elevated levels of blood inflammatory markers that carries high susceptibility to chronic morbidity, disability, frailty, and premature death. The pro-inflammatory state is characterized by high levels of pro-inflammatory markers, including IL-1, IL-6, IL-8, IL-13, IL-18, C-reactive protein, IFNα and IFNβ, TGFβ, TNF, and others. This overall imbalance towards persistent chronic inflammation results in insufficient tissue repair and tissue degeneration, which are characterized by increased susceptibility to aging-related diseases (including CVDs), decreased stress tolerance, the emergence of GSs, a worse response to treatment, and low functional capacity [26]. It is widely accepted that chronic, low-grade inflammation plays an important role in the pathogenesis of CVDs and atherosclerosis, independently of other cardiovascular risk factors [27]. Immune dysfunction and an inflammation-like pro-inflammatory state have been indicated in the pathogenesis of ACS [28,29], including cases of MI with non-obstructive coronary arteries (MINOCAs) [30,31]. Secondly, depression, which is considered one of the GSs and associated with previous MI according to our study, was also independently associated with a pro-inflammatory state and the progression of atherosclerotic disease, particularly in the carotid arteries [32]. Finally, inflammation plays a crucial role in the formation of blood clots [33,34]. Neutrophils can be detected in atheromatous plaques and may contribute to plaque formation and increased blood clot stability [35]. Apart from that, there are some data about the increase in clotting process markers in frailty patients without cardiovascular disease and diabetes [36]. The procoagulation status is also observed in patients after MI [37]. From this perspective, CVDs in older adults are consistent with the concept of compromised biological aging (i.e., a high basal level of inflammation associated with pathological phenotypic changes) [10].

## 6. Conclusions

Although this study showed an association between MI history and GSs, it is impossible to estimate the prognostic value of this association based on our study. The mechanisms and clinical implementation of the association between MI history and GSs should be investigated in future studies.

## Figures and Tables

**Table 1:** Demographic, anthropometric, and clinical characteristics according to MI history.

Parameter	All Patients (n = 4295)	MI History		*p*
		Yes (n = 540)	No (n = 3755)	
Age, years (M ± SD)	78.3 ± 8.4	80.3 ± 8.3	78.0 ± 8.4	**<0.001**
Male sex, %	29.7	59.8	28.2	**<0.001**
Height, m (M ± SD)	1.63 ± 0.09	1.63 ± 0.09	1.63 ± 0.09	0.896
Weight, kg (M ± SD)	73.9 ± 14.3	74.5 ± 14.9	73.8 ± 14.1	0.178
Body mass index, kg/m^2^ (M ± SD)	27.9 ± 5.0	28.0 ± 5.2	27.9 ± 4.9	0.759
Body mass, %				
Deficit	1.3	2.6	1.1	**0.004**
Normal	27.6	26.9	27.7	0.688
Overweight	40.9	39.5	41.1	0.487
Obesity	30.2	31.0	30.1	0.668
Degrees of obesity, % (n = 1264)				
I	72.2	73.9	71.9	0.585
II	21.6	19.4	22.0	0.451
III	6.2	6.7	6.1	0.783
Systolic BP, mm Hg (M ± SD)	136.1 ± 16.5	136.7 ± 17.7	136.0 ± 16.3	0.219
Systolic BP ≥ 140 mm Hg, %	38.9	44.3	38.1	**0.007**
Diastolic BP, mm Hg (M ± SD)	80.2 ± 9.5	80.5 ± 10.1	80.2 ± 9.5	0.367
Diastolic BP ≥ 90 mm Hg, %	18.1	22.2	17.5	**0.008**
Pulse BP, mm Hg (M ± SD)	55.9 ± 13.0	56.3 ± 13.9	55.8 ± 12.8	0.891
Heart rate, beats/min (M ± SD)	72.7 ± 8.6	73.0 ± 8.9	72.6 ± 8.5	0.620
Heart rate > 80 beats/min, %	13.8	15.4	13.5	0.238

Data in Bold: statistical significance.

**Table 2:** Results of CGA according to MI history.

Parameter		All Patients (n = 4295)	MI History		*p*
			Yes (n = 540)	No (n = 3755)	
SPPB, points *		6 (3; 9)	5 (2; 8)	6 (3; 9)	**<0.001**
Hand grip strength, kg *	Men	22 (16; 30)	20 (14; 28)	22 (16; 30)	**0.004**
	Women	16 (11;21)	12 (8;19)	16 (11;21)	**<0.001**
Decrease in hand grip strength, %		70.8	80.2	69.4	**<0.001**
Gait velocity, m/s *		0.60 (0.46;0.83)	0.57 (0.44;0.80)	0.63 (0.47;0.83)	**0.001**
Decrease in gait velocity, %		56.1	60.9	55.4	**0.024**
Mini-Cog test, points					**<0.001**
Me (25%; 75%)		3 (2; 4)	3 (1; 4)	3 (2; 4)	
M ± SD		2.89 ± 1.51	2.61 ± 1.50	2.94 ± 1.50	
Geriatric Depression Scale, points *		4 (2; 8)	6 (3; 10)	4 (2; 7)	**<0.001**
Basic Functional Activity Scale (Barthel Index), points					**<0.001**
Me (25%; 75%)		95 (85; 100)	95 (80; 100)	95 (85; 100)	
M ± SD		88.5 ± 17.8	84.3 ± 20.6	89.1 ± 17.3	
Lawton Instrumental Activities of Daily Living Scale, points *		7 (5; 8)	6 (4; 8)	7 (5; 8)	**<0.001**
MNA (Mini Nutritional Assessment) short-form, points *		12 (10; 13)	11.5 (10; 13)	12 (10; 13)	**<0.001**
Quality of life self-assessment according to VAS, points *		7 (5; 8)	6 (5; 7)	7 (5; 8)	**<0.001**
Health status self-assessment according to VAS, points					**<0.001**
Me (25%; 75%)		5 (5; 7)	5 (4; 6)	5 (5; 7)	
M ± SD		5.6 ± 2.0	4.9 ± 2.0	5.7 ± 2.0	
Pain self-assessment according to VAS, points *		3 (0; 5)	4 (0; 5)	3 (0; 5)	**<0.001**
Pain during the previous week self-assessment according to VAS, points *		4 (2; 6)	5 (3; 7)	4 (2; 6)	**<0.001**

* Results are presented as Me (25%; 75%). Data in Bold: statistical significance.

**Table 3:** Frequency of the use of assistive devices according to MI history.

Parameter	MI History		*p*
	Yes (n = 540)	No (n = 3755)	
Use of assistive devices, %	95.9	92.1	**0.001**
Number of assistive devices, Me (25%; 75%)	3 (2; 3)	2 (1; 3)	**<0.001**
Glasses/lens, %	82.6	78.8	**0.045**
Hearing aid, %	13.3	6.4	**<0.001**
Dentures, %	66.9	58.7	**<0.001**
Walking stick, %	45.6	30.8	**<0.001**
Crutches, %	3.7	2.2	**0.034**
Walkers, %	6.9	3.6	**<0.001**
Wheelchair, %	1.9	1.8	0.948
Orthopedic shoes, %	4.6	5.1	0.667
Orthopedic insoles, %	9.8	10.2	0.780
Orthopedic corset, %	5.9	4.5	0.152
Urological pads, %	16.9	13.3	**0.027**
Diapers/underpads, %	9.3	5.3	**<0.001**
Mobility aids (walking stick, crutches, walkers, wheelchair), %	50.6	34.0	**<0.001**
Absorbent clothing for urinary/fecal incontinence (urological pads, diapers), %	22.0	16.2	**0.001**

Data in Bold: statistical significance.

**Table 4:** Prevalence of geriatric syndromes according to MI history.

Parameter	N	MI History		*p*
		Yes (n = 540)	No (n = 3755)	
Chronic pain syndrome	4295	90.6	86.7	**0.013**
Impairment in basic activities of daily living	4295	71.9	59.6	**<0.001**
Impairment in instrumental activities of daily living	4295	71.9	51.7	**<0.001**
Frailty	4295	70.2	61.6	**<0.001**
Cognitive impairment	3537	69.1	59.5	**<0.001**
Depression	4271	57.9	46.7	**<0.001**
Urinary incontinence	4295	51.3	44.4	**0.002**
Falls during the previous year	4289	38.7	29.1	**<0.001**
Sensory deficit (any)	4294	19.1	14.9	**0.011**
Hearing loss	4292	14.6	11.4	**0.032**
Malnutrition	4295	9.6	5.4	**<0.001**
Visual impairment	4294	6.5	4.8	0.099
Orthostatic hypotension	3975	5.8	8.2	0.059
Fecal incontinence	4295	5.6	4.8	0.427
Bedsores	4293	3.9	2.0	**0.006**

Data in Bold: statistical significance.

**Table 5:** Associations between MI history and geriatric syndromes (univariate regression analysis).

Geriatric Syndromes	N	OR	95% CI	*p*
Urinary incontinence	4295	1.32	1.10–1.58	**0.002**
Sensory deficit	4294	1.33	1.02–1.72	**0.032**
Hearing loss	4292	1.35	1.07–1.70	**0.012**
Chronic pain syndrome	4295	1.47	1.08–1.99	**0.013**
Frailty	4295	1.47	1.20–1.78	**<0.001**
Cognitive impairment	3537	1.52	1.24–1.87	**<0.001**
Falls during the previous year	4289	1.53	1.27–1.85	**<0.001**
Depression	4271	1.57	1.31–1.89	**<0.001**
Impairment in basic activities of daily living	4295	1.73	1.42–2.11	**<0.001**
Malnutrition	4295	1.87	1.36–2.58	**<0.001**
Bedsores	4293	1.96	1.20–3.20	**0.007**
Impairment in instrumental activities of daily living	4295	2.38	1.95–2.90	**<0.001**

Note: dependent variable: geriatric syndromes. Data in Bold: statistical significance.

**Table 6:** Associations between MI history and geriatric syndromes (multivariate regression analysis, n = 3516).

Predictors	OR	95% CI	*p*
Impairment in basic activities of daily living	1.28	1.00–1.63	**0.047**
Depression	1.28	1.03–1.58	**0.026**
Falls during the previous year	1.45	1.18–1.79	**<0.001**
Impairment in instrumental activities of daily living	1.86	1.47–2.36	**<0.001**
Male sex	1.89	1.53–2.33	**<0.001**

Note: dependent variable: MI history. Data in Bold: statistical significance.

## Data Availability

Data presented in this study are available upon reasonable request.
